# Preoperative PARP Inhibitors in Ovarian Cancer Trials: Connecting Molecular Oncology and Cytoreductive Surgery

**DOI:** 10.3390/cancers18132157

**Published:** 2026-07-05

**Authors:** Cezary Miedziarek, Paweł Caputa, Hubert Bochyński, Mikołaj Piotr Zaborowski, Ewa Nowak-Markwitz

**Affiliations:** 1 Division of Gynecological Oncology, Department of Gynecology, Poznan University of Medical Sciences, 61-701 Poznań, Poland; pcaputa@ump.edu.pl (P.C.); hbochynski@ump.edu.pl (H.B.); mzaborowski@ump.edu.pl (M.P.Z.);; 2 Doctoral School, Poznan University of Medical Sciences, 61-701 Poznań, Poland; 3 Institute of Bioorganic Chemistry, Polish Academy of Sciences, 61-772 Poznań, Poland

**Keywords:** ovarian cancer, PARP inhibitors, cytoreductive surgery, neoadjuvant therapy, complete cytoreduction

## Abstract

Cytoreductive surgery remains one of the key treatment modalities in advanced ovarian cancer. Complete cytoreduction is the main surgical goal. PARP inhibitors are currently established mainly as maintenance therapy after response to platinum-based chemotherapy, particularly in patients with *BRCA*-mutated or homologous recombination-deficient tumors. Their use before cytoreductive surgery remains investigational. This review evaluates preoperative PARP inhibition from a surgical perspective. This narrative review summarizes current evidence, ongoing clinical trials, and perioperative considerations related to preoperative or neoadjuvant PARP inhibitor strategies in advanced ovarian cancer. Particular attention was given to the review of current clinical trials’ strategies, resectability, complete cytoreduction, patient selection, perioperative safety, treatment timing, and surgery-specific endpoints. Current studies explore several preoperative approaches, including short window-of-opportunity treatment before primary debulking surgery, PARP inhibitor monotherapy as potential conversion therapy in homologous recombination-deficient disease, PARP inhibitor-based strategies before interval debulking surgery, combination regimens with immunotherapy or antiangiogenic therapy, and preoperative PARP inhibitor use before secondary cytoreduction in recurrent disease. These studies suggest that preoperative PARP inhibition may provide biological and surgical insights, but available evidence remains preliminary. Key concerns include hematologic toxicity, surgical postponement, perioperative complications, wound healing, postoperative recovery, and the risk of delaying standard chemotherapy or surgery. Preoperative PARP inhibitor therapy is theoretically promising but an unproven strategy in ovarian cancer. Its future value will depend on prospective trials showing that it can safely improve resectability and complete cytoreduction without compromising treatment timing. Future studies should include surgery-specific endpoints in addition to conventional oncologic outcomes.

## Simple Summary

PARP inhibitors are already widely used after chemotherapy as maintenance treatment in patients with advanced ovarian cancer, especially in *BRCA*-mutated tumors or those with homologous recombination deficiency. However, their possible use before cytoreductive surgery remains experimental. This review discusses whether preoperative PARP inhibitor treatment could help surgeons by reducing tumor burden, improving the likelihood of complete tumor removal, and supporting better patient selection for primary, interval, or secondary cytoreductive surgery. Current early-phase and ongoing studies suggest that this approach is theoretically promising but also raise important safety concerns, including blood count abnormalities, surgical delays, wound-healing complications, and postoperative recovery. Future trials should assess not only cancer response and survival but also surgical outcomes, including complete cytoreduction, perioperative complications, transfusion requirements, and time to further treatment.

## 1. Introduction

### 1.1. Clinical Background

Cytoreductive surgery remains the most important factor of treatment for advanced epithelial ovarian cancer [1,2]. In high-grade serous ovarian cancer, most patients present with advanced-stage disease because early symptoms are nonspecific and clinically silent progression is common [3]. Debulking surgery is therefore performed to remove all visible tumor deposits, as complete macroscopic cytoreduction remains one of the strongest prognostic factors and a determinant of subsequent treatment strategy [3,4]. In FIGO stage III–IV disease, the choice between primary debulking surgery (PDS) and neoadjuvant chemotherapy (NACT) followed by interval debulking surgery (IDS) depends on disease distribution, patient clinical condition, operative risk, likelihood of complete macroscopic resection, and geographic or institutional factors, including hospital expertise and surgeon preference [3,5,6]. PDS is preferred when complete resection is feasible with acceptable morbidity, whereas NACT is recommended when upfront complete cytoreduction is unlikely or unsafe [3,5]. The surgical aim remains removal of all visible disease, which is one of the strongest prognostic factors in advanced ovarian cancer [3,5,7]. Resectability assessment should integrate imaging, disease distribution, performance status, comorbidities, nutritional condition, and use of scoring systems [3,5,8]. Diagnostic laparoscopy may be useful in selected patients with extensive peritoneal disease, as laparoscopic triage has been shown to reduce futile laparotomies and improve selection for PDS [9,10].

### 1.2. Rationale for Preoperative PARP Inhibition

PARP inhibitors are currently established mainly as maintenance therapy after response to platinum-based chemotherapy in advanced ovarian cancer, with the greatest benefit observed in patients with *BRCA1/2*-mutated or homologous recombination-deficient (HRD) tumors [3,11,12]. A summary of the most important currently available PARP inhibitors and their adverse event profiles is presented in Table 1. Their use in this setting is biologically supported by the concept of synthetic lethality, as HRD cancer cells are particularly vulnerable to PARP inhibition [13,14]. The rationale for earlier preoperative use is that selected *BRCA*-mutated or HRD-positive tumors may already be sensitive to PARP inhibitors prior to cytoreductive surgery. Preclinical and ex vivo data support the biological plausibility of PARP inhibitor monotherapy in selected ovarian cancers. Patient-derived organoids and spheroid models have shown sensitivity to olaparib or niraparib, particularly in tumors with *BRCA* alterations or functional HRD assessed by DNA repair assays [15,16,17]. These findings suggest that PARP inhibitors may have activity without concurrent DNA-damaging chemotherapy in molecularly selected models. This could theoretically reduce tumor burden, improve resectability, and provide a direct biological assessment of tumor response before major surgery. This concept is now being explored in ongoing or early-phase studies [18,19,20,21].

### 1.3. Knowledge Gap

**Figure 1:**
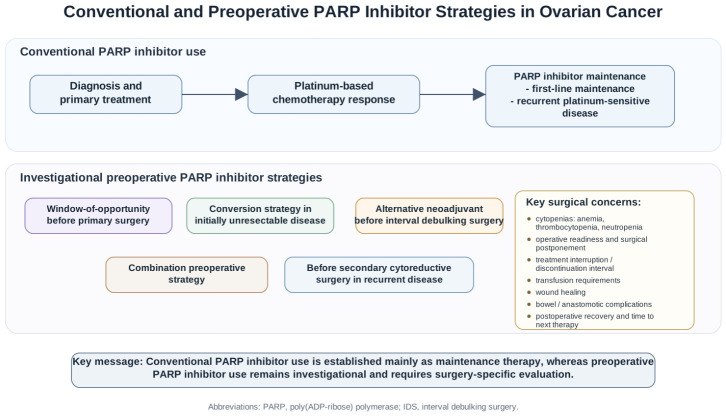
Potential strategies for preoperative PARP inhibitor use in ovarian cancer from a surgical perspective.

Existing literature on PARP inhibitors in ovarian cancer has primarily focused on maintenance therapy, molecular selection, mechanisms of synthetic lethality, and systemic oncologic outcomes. In contrast, the specific surgical implications of administering PARP inhibitors before cytoreductive surgery remain insufficiently defined. The scarcity of early preoperative PARP inhibitor trials probably reflects ethical, clinical, and logistical barriers. Standard PDS or platinum-based NACT cannot be delayed without strong justification. Preoperative PARP inhibition could theoretically be integrated into the surgical pathway at several points. The most conservative model is a short window-of-opportunity strategy before PDS, designed primarily to assess feasibility, safety, and early biological response without delaying surgery [20]. A second model is neoadjuvant PARP inhibitor therapy as conversion therapy in selected *BRCA*-mutated or HRD-positive patients with initially unresectable disease, in which the relevant surgical outcome would be conversion to operability and complete cytoreduction [18,19]. PARP inhibitors may also be studied as an alternative to, or in combination with, standard platinum-taxane NACT before IDS [21]. Finally, preoperative PARP inhibitors have been explored before secondary cytoreductive surgery in platinum-sensitive recurrent ovarian cancer, where response to PARP inhibitors may help precisely assess tumor biology [22] (Figure 1). Across these scenarios, the central surgical question is whether PARP inhibitors can safely improve resectability and complete cytoreduction without compromising treatment timing. This review evaluates preoperative use of PARP inhibitors in ovarian cancer from a surgical perspective, with particular focus on resectability, complete cytoreduction, surgical candidacy, perioperative safety, timing of surgery, and surgical endpoints for future clinical trials.

**Table 1:** Selected PARP inhibitors, representative clinical trials, and grade ≥ 3 hematologic adverse events reported in ovarian cancer studies.

PARP Inhibitor	Selected Ovarian Cancer Trials	Selected Non-Ovarian Trials	Hematologic AEs in OC Trials, Grade ≥ 3
Olaparib [11,23,24,25,26,27,28]	SOLO-1; SOLO-2; Study 19; PAOLA-1	Breast: Phase II *BRCA*-mutated advanced breast cancer proof-of-concept trial, OlympiA. Pancreatic: POLO. Prostate: PROfound, PROpel.	Decreased hemoglobin/anemia 13–21% Decreased ANC/neutropenia 6–9% Decreased plate-lets/thrombocytopenia 1–2.4% * Monotherapy and combination therapy data
Niraparib [12,29,30,31,32,33]	NOVA; PRIMA; NORA; PRIME	Prostate: MAGNITUDE, AMPLITUDE. Breast/TNBC: BRAVO, TOPACIO-TNBC.	Decreased hemoglobin/anemia 18–33% Decreased ANC/neutropenia 13–23% Decreased platelets/thrombocytopenia 14–38% * Monotherapy data
Rucaparib [34,35,36,37,38,39]	ARIEL2; ARIEL3 updated safety; ARIEL4.	Prostate: TRITON2, TRITON3. Pancreatic: RUCAPANC.	Decreased hemoglobin/anemia 13–29% Decreased ANC/neutropenia 6–11% Decreased platelets/thrombocytopenia 2–6% * Monotherapy data
Talazoparib [40,41,42,43,44,45,46]	Limited early-phase ovarian data.	Breast: ABRAZO, EMBRACA. Prostate: TALAPRO-1, TALAPRO-2, TALAPRO-3.	N/A
Pamiparib [47,48,49,50]	BGB-290-102	Advanced solid tumors; early studies include gastric and SCLC cohorts.	Decreased hemoglobin/anemia 41.6% Decreased ANC/neutropenia 33.6% Decreased platelets/thrombocytopenia 4.4% * Monotherapy data
Fuzuloparib [51,52,53,54,55]	FZOCUS-2; FZOCUS-1; FZOCUS-3.	Prostate: FUZUPRO. Pancreatic: ongoing trials	Decreased hemoglobin/anemia 25.1–31.0% Decreased ANC/neutropenia 12.6–12.8% Decreased plate-lets/thrombocytopenia 16.8–28.7% * Monotherapy and combination therapy data
Senaparib [56,57]	FLAMES.	Early-stage trials	Decreased hemoglobin/anemia 29% Decreased ANC/neutropenia 25% Decreased platelets/thrombocytopenia 27% * Monotherapy data
Veliparib [58,59,60]	VELIA.	Breast: BROCADE3. Pancreatic: veliparib plus platinum/gemcitabine-based studies.	Decreased hemoglobin/anemia 38–41% Decreased ANC/neutropenia 58–62% Decreased platelets/thrombocytopenia 28–31% * Combination therapy data

* Data characteristics.

## 2. Methods

This narrative review was based on a targeted literature search of PubMed/MEDLINE, ClinicalTrials.gov, and relevant clinical trial registry records. Additional sources included published study protocols, conference abstracts, and current clinical guidelines when directly relevant to preoperative or neoadjuvant PARP inhibitor strategies in ovarian cancer. The search included studies available up to April 2026. Search terms included combinations of “ovarian cancer,” “high-grade serous ovarian cancer,” “PARP inhibitor,” “olaparib,” “niraparib,” “rucaparib,” “neoadjuvant,” “preoperative,” “window of opportunity,” “cytoreductive surgery,” “interval debulking surgery,” and “secondary cytoreduction”. Studies were selected if they addressed PARP inhibitor use before cytoreductive surgery and neoadjuvant PARP inhibitor strategies in ovarian cancer patients. Because mature clinical evidence in this field remains limited, trial protocols, registry entries, and conference abstracts were included when they provided information on ongoing or emerging strategies.

## 3. Current Clinical Trials and Approaches for Preoperative PARP Inhibitor Therapy

### 3.1. Overview of Ongoing and Early-Phase Studies

The use of PARP inhibitors before cytoreductive surgery remains investigational, but several ongoing or early-phase studies provide a framework for discussing this strategy from a surgical perspective. These studies differ in clinical context: some evaluate a short preoperative “window-of-opportunity” before primary debulking surgery, others assess PARP inhibitors as neoadjuvant treatment before IDS, and some explore PARP inhibition before secondary cytoreduction in recurrent disease [18,19,20,21,61,62,63]. Current guidelines still define PDS or NACT followed by IDS as the standard initial approach for advanced ovarian cancer [3,5]. Therefore, preoperative PARP inhibitor treatment should still be considered as a trial strategy rather than routine care. A summary of ongoing trials identified through ClinicalTrials.gov and relevant literature search that use different PARP inhibitor approaches is shown in Table 2.

### 3.2. “Window-of-Opportunity” Studies Before PDS

“Window-of-opportunity” studies represent the most conservative model of preoperative PARP inhibitor treatment. In this approach, a PARP inhibitor is administered for a short period between diagnosis and planned PDS. The main goals are to evaluate feasibility, safety, and early biological response. The AGO-OVAR 27/WoO trial is an example of this approach, evaluating olaparib alone or in combination with durvalumab before primary debulking surgery in patients with high-grade epithelial ovarian cancer [20]. The surgical relevance of this model lies in whether preoperative PARP inhibitors can be safely introduced without delaying PDS or compromising perioperative outcomes. Similar studies may also be beneficial from a molecular perspective, as they can provide paired tissue samples before and after PARP inhibitor exposure, allowing assessment of the DNA damage response, homologous recombination function, proliferation, changes in the immune microenvironment, and early resistance mechanisms. However, because the treatment window is short, these trials are more likely to inform biological feasibility than to demonstrate meaningful tumor downsizing or improved resectability.

### 3.3. PARP Inhibitors as Conversion Therapy Before Cytoreductive Surgery

Another strategy is the use of PARP inhibitors as a primary therapy in patients with initially unresectable disease. The NANT study evaluates niraparib as neoadjuvant monotherapy in advanced ovarian cancer with HRD and was designed to assess both efficacy and safety in a preoperative setting [18,19]. Neoadjuvant niraparib monotherapy has shown antitumor activity and favorable surgical outcomes, including a promising objective response rate and R0 resection rate, with acceptable toxicity in patients with HRD-positive unresectable high-grade serous ovarian cancer. These findings suggest that niraparib may represent a potential investigational alternative for selected patients who are unwilling or unable to receive standard NACT [64].

### 3.4. PARP Inhibitors Versus Standard Neoadjuvant Chemotherapy Before IDS

Another approach is to directly compare PARP inhibitor-based neoadjuvant therapy with standard platinum-taxane chemotherapy before IDS. The OPAL-C cohort study compared neoadjuvant niraparib with platinum-taxane doublet chemotherapy in patients with HRD, FIGO stage III–IV ovarian cancer who are candidates for neoadjuvant treatment followed by IDS [21,61]. This trial design was relevant from a surgical perspective because it directly tested whether a molecularly targeted neoadjuvant approach could compete with standard platinum-taxane chemotherapy in preparing patients for IDS. However, the study was stopped after meeting the prespecified futility criteria, indicating a low probability that the experimental strategy would demonstrate the expected clinical benefit if the trial were to be completed [21]. This finding is important because it highlights that replacing standard neoadjuvant chemotherapy with PARP inhibitor-based treatment is unlikely, even in a molecularly selected population.

### 3.5. PARP Inhibitor-Based Combinations Before Surgery

PARP inhibitor-based combinations before surgery are also being explored. NEOCATS evaluates olaparib in combination with durvalumab and bevacizumab in newly diagnosed advanced ovarian cancer, with treatment administered before IDS in responding patients [62]. The AGO-OVAR 27/WoO study also includes an olaparib plus durvalumab cohort before PDS [20]. These approaches are biologically attractive because PARP inhibitors can be combined with immune checkpoint inhibitors and antiangiogenic therapy. The main surgical concern is potential toxicity. PARP inhibitors may cause anemia, thrombocytopenia, and neutropenia [65,66,67]. Bevacizumab may affect wound healing and gastrointestinal safety [68,69], and immune checkpoint inhibitors may cause immune-related adverse events [70]. Therefore, combination strategies should be assessed using specific perioperative endpoints, including surgical delay, preoperative blood counts, transfusion rates, wound complications, bowel complications, and 30- and 90-day morbidity [71,72,73,74,75].

### 3.6. PARP Inhibition Before Secondary Cytoreductive Surgery

Preoperative PARP inhibition has also been explored in recurrent ovarian cancer before secondary cytoreductive surgery. The NEO trial investigated neoadjuvant olaparib in patients with platinum-sensitive relapsed high-grade serous ovarian cancer eligible for surgery [63]. Cytoreductive surgery depends strongly on the ability to achieve complete resection. In this context, the NEO trial suggests that neoadjuvant olaparib followed by cytoreductive surgery is feasible and safe in patients with platinum-sensitive recurrent high-grade serous ovarian cancer. Among patients with resectable disease at secondary cytoreduction, postoperative olaparib alone appeared comparable to chemotherapy followed by olaparib. It was associated with lower toxicity, suggesting a potential chemotherapy-free approach in this highly selected population [63]. From a surgical perspective, these findings are relevant because preoperative PARP inhibition may help identify tumors with favorable DNA repair vulnerability and less aggressive biology before operative intervention. Ongoing translational analyses may further clarify biomarkers of response and resistance, as the primary outcomes include differences in levels of PAR or PARP-1 before and after study treatment and the assessment of mutations in *BRCA1/2*, *RAD51B*, *RAD51C*, *RAD51D*, *PPM1D*, *FANCM*, *BRIP1*, *PALB2*, and *BARD1* in germline tissue compared to tumor tissue. However, these results should be interpreted cautiously. They cannot be directly extrapolated to the first-line setting, where patients are treatment-naive or minimally treated and where the standard pathway remains PDS or platinum-based NACT followed by IDS.

## 4. Perioperative Safety and Timing of Surgery

Preoperative PARP inhibition raises a clinical problem: toxicities that are usually manageable in the maintenance setting may become clinically relevant when a patient is being prepared for extensive cytoreductive surgery. In this context, anemia, thrombocytopenia, neutropenia, treatment interruptions, transfusion requirements, wound healing, and postoperative recovery are not only adverse events but also potential determinants of whether surgery can be performed safely and on time.

### 4.1. Hematologic Toxicity and Operative Readiness

Hematologic toxicity is the most important perioperative concern with preoperative PARP inhibition. In the maintenance setting, dose interruption or reduction is an acceptable approach [76,77]. Before cytoreductive surgery, the same toxicity may lead to delay of surgery, reduced operative readiness, transfusion, or a change in treatment strategy. This is particularly relevant because cytoreductive surgery for advanced ovarian cancer may involve bowel resection, diaphragmatic stripping, splenectomy, liver mobilization, peritonectomy, and other procedures associated with potential massive blood loss and postoperative infectious risk [72,78,79]. Each cytopenia has distinct surgical implications. Anemia may reduce physiologic reserve before long operations, increase the likelihood of perioperative transfusion, and complicate recovery after multivisceral resection. Olaparib commonly causes anemia, leading to dose interruption or reduction. In SOLO-1, anemia was the most frequent adverse reaction leading to interruption or reduction. In PAOLA-1, anemia was also the most frequent reason for such interventions in the olaparib-bevacizumab arm [11,23]. In a preoperative trial, hemoglobin decline should therefore be recorded not only as an adverse event, but as a surgical readiness variable. Severe thrombocytopenia is particularly important for niraparib. In the PRIMA trial, thrombocytopenia emerged as one of the most characteristic and clinically relevant toxicities [12]. This is particularly important in the preoperative setting, because platelet decline may directly affect operative readiness, increase bleeding risk, and potentially delay cytoreductive surgery [80,81]. Thus, when niraparib is considered before surgery, thrombocytopenia should be monitored not only as a treatment-related adverse event but also as a perioperative safety variable. Although individualized starting doses reduce this risk [82], thrombocytopenia remains a key barrier to safe major surgery. Neutropenia is perioperatively relevant because extensive ovarian cancer surgery often involves bowel surgery, long operative time, drains, and postoperative collections. Future preoperative studies should therefore report absolute neutrophil count immediately before surgery, postoperative infections, abscesses, anastomotic complications, and antibiotic use [83,84,85,86].

### 4.2. Transfusion Risk and Surgical Postponement

In standard maintenance therapy, transfusion is usually treated as a supportive intervention. In preoperative PARP inhibitor strategies, the surgical endpoint should be considered. Preoperative anemia may increase the need for red blood cell transfusion before or during cytoreduction, while thrombocytopenia may require platelet transfusion or delay surgery. In general surgical practice, platelet transfusion is usually recommended when the platelet count is below 50 × 10^9^/L before major non-neuraxial surgery. At the same time, higher thresholds may be required for procedures with particularly high bleeding risk or critical-site surgery [80,87,88]. In contrast, niraparib prescribing information addresses thrombocytopenia mainly from the drug-safety perspective: treatment should be withheld when platelet count falls below 100,000/µL, resumed at a reduced dose after recovery, and platelet transfusion should be considered at ≤10,000/µL, or at higher platelet counts when additional bleeding risk factors, such as anticoagulant or antiplatelet therapy, are present [89,90]. Therefore, in patients receiving niraparib before cytoreductive surgery, clinicians should recognize that the threshold for intervention may need to be substantially higher than the threshold used to prevent spontaneous bleeding, because major abdominal surgery requires adequate hemostatic reserve. For future trials, transfusion-related outcomes should be reported, taking into account the following parameters: baseline hemoglobin and platelet count, minimum values during preoperative treatment, number of patients requiring transfusion before surgery, intraoperative transfusion rate, postoperative transfusion rate, and surgery postponement due to cytopenia. These data would allow evaluation of whether preoperative PARP inhibitor therapy prepares patients for surgery or creates new perioperative obstacles.

### 4.3. Timing of PARP Inhibitor Interruption Before Surgery

Given the novelty of the described approach, there is no well-established evidence-based interval for discontinuing PARP inhibitors before cytoreductive surgery. In maintenance therapy, drug interruption is typically guided by toxicity, recurrence, or scheduled end of treatment [11,23,91]. In the preoperative setting, interruption must also account for operative timing, expected blood loss, wound healing, and recovery of bone marrow reserve. A practical trial-based approach would be to define a preoperative discontinuation window and require the fulfillment of predefined hematologic criteria before surgery. These criteria should include hemoglobin, platelet count, neutrophil count, absence of uncontrolled infection, and recovery from clinically relevant non-hematologic toxicity. Trials should report the interval between the last PARP inhibitor dose and surgery, the proportion of patients whose surgery was delayed because of toxicity, and the proportion who required dose interruption before the planned surgical date.

### 4.4. Postoperative Recovery and Time to Systemic Treatment

The perioperative impact of preoperative PARP inhibitor therapy should also be assessed after surgery. The key issue is whether preoperative treatment compromises postoperative recovery or delays the next treatment step. In ovarian cancer, prolonged postoperative recovery may delay chemotherapy, maintenance therapy, or trial-defined postoperative treatment, which could diminish any preoperative benefit [92,93,94,95,96]. Relevant postoperative endpoints should include length of hospital stay, intensive care admission, reoperation, readmission, Clavien–Dindo complications, 30- and 90-day morbidity, postoperative infections, wound complications, thromboembolic events, and time from surgery to postoperative systemic therapy [71,97,98,99]. Importantly, these outcomes should be interpreted in light of the complexity of the surgery.

### 4.5. Safety Concerns with PARP Inhibitor-Based Combinations

Combination strategies have additional perioperative concerns. PARP inhibitors combined with chemotherapy may intensify bone marrow suppression, which is directly relevant before interval debulking surgery [100,101,102]. PARP inhibitors combined with immune checkpoint inhibitors introduce the possibility of immune-related adverse events, including pneumonitis, hepatitis, colitis, endocrinopathies, or steroid exposure before surgery [103,104,105]. PARP inhibitors combined with bevacizumab are particularly important surgically because bevacizumab may impair wound healing and increase the risk of gastrointestinal perforation or anastomotic complications [106,107]. Literature commonly recommends withholding bevacizumab for 4–8 weeks before major surgery and restarting no earlier than 28 days postoperatively, after adequate wound healing [108,109,110]. Therefore, any neoadjuvant regimen combining PARP inhibitors with bevacizumab must define the interval between the last bevacizumab dose and cytoreductive surgery, especially if bowel resection or anastomosis is anticipated.

## 5. Patient Selection for Neoadjuvant PARP Inhibitor Strategies

Based on current surgical standards, PARP inhibitor biology, ongoing neoadjuvant trial designs, and known hematologic toxicity profiles, the most rational candidates for surgery-oriented neoadjuvant PARP inhibitor trials would be clinically stable patients with newly diagnosed advanced high-grade serous ovarian, fallopian tube, or primary peritoneal cancer, confirmed *BRCA1/2* mutation or HRD-positive status, low probability of primary R0 resection, lack contraindications for combination drugs, and adequate hematologic reserve [3,5,18,23,61,64,91,111]. The proportion of patients who might meet all criteria for preoperative PARP inhibitor trials is likely substantially smaller than the proportion of patients with *BRCA*-mutated or HRD-positive HGSOC. Although approximately 50–60% of high-grade serous ovarian cancers may be HRD-positive, eligibility would be further restricted by unresectability on surgical assessment, clinical stability, adequate hematologic reserve, absence of urgent symptoms, and rapid availability of molecular testing [112]. Molecular diagnosis is necessary because the biological rationale for PARP inhibitors is strongest in *BRCA*-mutated and HRD-positive tumors, and genetic test results are decisive for selecting the appropriate drug [13]. Future patient selection should also move beyond static *BRCA1/2* and genomic HRD status. RAD51 foci formation is a functional marker of homologous recombination competence and may help identify tumors with restored HR function despite genomic HRD features [15,16]. SLFN11 has been associated with sensitivity to DNA-damaging agents and PARP inhibition in several tumor models and may represent another candidate biomarker of replication stress vulnerability [113,114]. KELIM, based on CA-125 elimination kinetics during chemotherapy, does not directly measure PARP inhibitor sensitivity, but may provide dynamic information on tumor chemosensitivity and early treatment response [115]. Other potentially relevant tools include ctDNA dynamics, detection of *BRCA* reversion mutations, and patient-derived organoid assays [15,116]. These biomarkers remain investigational, but they may help refine the selection of patients for future preoperative PARP inhibitor trials. Histologically, high-grade serous carcinoma represents the most appropriate target subtype because it is most closely associated with *BRCA* mutations, HRD, platinum sensitivity, and PARP inhibitor responsiveness [3,117]. From a surgical perspective, preoperative PARP inhibition should be investigated primarily in patients for whom it could realistically alter management, such as those with initially unresectable disease, rather than in patients already suitable for straightforward primary complete cytoreduction. Candidates should also be clinically stable, without bowel obstruction, impending perforation, uncontrolled infection, rapidly progressive symptomatic disease, severe malnutrition, or urgent need for immediate surgery or chemotherapy [5,118]. Adequate marrow reserve is essential because anemia, thrombocytopenia, and neutropenia may delay cytoreductive surgery or increase perioperative risk [71,87]. Patients with HR-proficient or unknown molecular status, inadequate tissue for rapid testing, poor clinical stability, urgent surgical complications, or baseline cytopenias are unlikely to be suitable candidates outside clinical trials. Overall, preoperative PARP inhibition should currently be viewed as a narrowly selected investigational strategy for molecularly defined, surgically challenging but clinically stable patients, to improve operability and complete cytoreduction without compromising timely standard treatment. Future neoadjuvant PARP inhibitor strategies should also account for treatment sequencing and logistical aspects of molecular testing. This approach is clinically feasible only when *BRCA* or HRD results are available alongside a histopathological diagnosis, enabling molecularly guided treatment without delaying standard surgery or platinum-based chemotherapy; empirical short-term PARP inhibition until molecular results are available would require strict trial-based assessment. Chemotherapy may be less effective after progression on PARP inhibitors [119,120]. Future studies should also evaluate whether neoadjuvant PARP inhibition affects subsequent platinum-taxane response, postoperative treatment sensitivity, and mechanisms of cross-resistance.

## 6. Possible Reasons for Uncertain Success of Preoperative PARP Inhibitor Strategies

Several factors may explain why preoperative PARP inhibitor strategies have shown limited or uncertain success so far. First, PARP inhibitor monotherapy may not induce sufficiently rapid or consistent tumor shrinkage to replace platinum-taxane neoadjuvant chemotherapy in patients with high-volume advanced ovarian cancer. This is supported by OPAL-C, which compared niraparib with platinum-taxane neoadjuvant chemotherapy in HRD-positive FIGO stage III-IV ovarian cancer and was stopped after meeting prespecified futility criteria [21,61]. Second, genomic HRD positivity does not always indicate functional sensitivity to PARP inhibition, as resistance may result from homologous recombination restoration, *BRCA* reversion mutations, replication fork stabilization, or tumor heterogeneity [116,121,122]. Third, a biological response may not necessarily translate into improved resectability or complete macroscopic resection. Finally, hematologic toxicity and logistical requirements, including rapid molecular testing and surgical scheduling, may limit the feasibility of this strategy in the preoperative setting [65,76,77].

## 7. Conclusions and Future Directions

Preoperative use of PARP inhibitors remains a theoretically promising but investigational strategy in ovarian cancer, with the strongest potential in selected patients with *BRCA* mutation or HRD. Its potential role should be assessed not only by antitumor activity, but also by its ability to improve resectability, support complete cytoreduction, and maintain safe surgical timing. Future trials should evaluate different PARP inhibitors, both as monotherapy and in combination, across clearly defined surgical settings, including primary, interval, and secondary cytoreduction. Importantly, these studies should include surgery-specific endpoints in addition to conventional oncologic outcomes, including complete cytoreduction, conversion to resectability, surgical complexity, perioperative morbidity, transfusion requirements, postoperative recovery, and time to further systemic treatment. Beyond conventional chemotherapy, antiangiogenic therapy, and immunotherapy, several emerging rational combinations may be relevant for future preoperative PARP inhibitor strategies. Inhibitors of DNA damage response pathways, including ATR, CHK1, WEE1, DNA-PK, and POLQ inhibitors, may enhance replication stress, impair homologous recombination repair, or overcome PARP inhibitor resistance [123,124,125,126]. Epigenetic approaches, such as HDAC or BET inhibition, may also sensitize tumors to PARP inhibition by downregulating DNA repair pathways and inducing a “*BRCA*-like” phenotype [127,128]. Antibody-drug conjugates carrying DNA-damaging agents may represent another future strategy, potentially allowing more selective delivery of cytotoxic injury while avoiding some of the overlapping toxicity observed with conventional chemotherapy combinations [129]. However, all of these approaches remain investigational in the preoperative ovarian cancer setting. Future trials should determine whether such combinations can improve tumor response and resectability without compromising hematologic reserve, surgical timing, wound healing, or postoperative recovery. Until prospective data demonstrate clear surgical benefit and acceptable perioperative safety, preoperative PARP inhibition should remain investigational and should currently be limited to clinical trial settings.

## Figures and Tables

**Table 2:** Current clinical trials of preoperative or neoadjuvant PARP inhibition and their surgical relevance.

Trial/Identifier	Evidence Type	Study Status	Population	PARP Inhibitor Strategy	Surgical Setting	Primary Outcomes
NANT/NCT04507841 [18,19]	Prospective, interventional, single-arm, open-label, phase II study	Active, not recruiting	Newly diagnosed advanced ovarian cancer with homologous recombination deficiency; patients considered unsuitable for optimal primary cytoreduction	Niraparib monotherapy as neoadjuvant therapy in patients with advanced ovarian cancer, primary peritoneal cancer, fallopian tube cancer (FIGO stage III or IV)	Patients with low likelihood of achieving R0 cytoreduction by imaging assessment or laparoscopic evaluation, or cannot tolerate PDS due to poor conditions	R0 resection rate after neoadjuvant niraparib; objective response rate after neoadjuvant treatment.
AGO-OVAR 27/WoO/NCT04644289 [20]	Multi-center, prospective, open-label, phase II trial	Recruiting	Histologically proven high-grade epithelial ovarian cancer planned for primary debulking surgery	Olaparib alone or olaparib plus durvalumab before surgery	Short preoperative exposure (max. 28 days) before primary debulking surgery	Successful completion of window-of-opportunity treatment, defined by adequate dose intensity, absence of treatment-related surgical delay, no clinical progression before surgery, and no toxicity interfering with perioperative management.
OPAL-C/NCT06964165 [21,61]	Open-label Phase 2, Randomized, Controlled Multicenter Study	Terminated (the study stopped after meeting pre-specified futility criteria)	Homologous recombination-deficient FIGO stage III–IV ovarian cancer	Niraparib compared with Carboplatin + Paclitaxel neoadjuvant chemotherapy	Neoadjuvant treatment before interval debulking surgery	Pre-IDS unconfirmed objective response rate according to investigator-assessed RECIST v1.1.
NEOCATS/NCT06650709 [62]	Single-arm, proof-of-concept phase 2 study.	Not yet recruiting (scheduled to begin on 1 November 2024)	Suspected or confirmed diagnosis of high-grade serous ovarian cancer who are not thought to be candidates for primary debulking surgery and are considered candidates for neoadjuvant chemotherapy and are not known to have *BRCA*-mutation-associated HGSOC	Olaparib plus durvalumab plus bevacizumab. Patients will receive the following triplet therapy for 2 cycles: Olaparib continuous dosing; Durvalumab on day 1; and Bevacizumab on days 1 and 15 of a 28-day cycle.	Patients who respond to therapy after 2 cycles will receive 1 further cycle, followed by interval cytoreductive surgery planned 3–4 weeks after completion of cycle 3 and at least 28 days after the last dose of Bevacizumab.	Objective response rate after neoadjuvant triplet therapy according to investigator-assessed RECIST v1.1.
NEO/NCT02489006 [63]	Phase II, Open-Label, Randomized, Multi-Centre Study	Active, not recruiting	Platinum-sensitive recurrent high-grade serous ovarian cancer is eligible for secondary cytoreductive surgery	Olaparib before secondary cytoreduction	Preoperative treatment before secondary cytoreductive surgery. Response rate to olaparib in the neoadjuvant period as a secondary outcome.	Response to neoadjuvant olaparib before secondary cytoreductive surgery; translational assessment of PAR/PARP-1 modulation and DNA repair gene alterations.

Abbreviations: *BRCA*, breast cancer gene; FIGO, International Federation of Gynecology and Obstetrics; PARP, poly(ADP-ribose) polymerase; WoO, window of opportunity.

## Data Availability

No new data were created or analyzed in this study. Data sharing is not applicable to this article.

## Abbreviations

The following abbreviations are used in this manuscript:

AGO	German Gynecological Oncology Group
AGO-OVAR	Ovarian Cancer Study Group of the German Gynecological Oncology Group
BARD1	*BRCA1*-associated RING domain protein 1
*BRCA*	Breast cancer gene
*BRCA1/2*	Breast cancer gene 1/2
BRIP1	*BRCA1*-interacting helicase 1
CC BY	Creative Commons Attribution
ctDNA	Circulating tumor DNA
EOC	Epithelial ovarian cancer
FANCM	FA complementation group M
FIGO	International Federation of Gynecology and Obstetrics
HR	Homologous recombination
HRD	Homologous recombination deficiency
IDS	Interval debulking surgery
KELIM	CA-125 elimination rate constant K
NACT	Neoadjuvant chemotherapy
NANT	Neoadjuvant niraparib therapy
NCT	National Clinical Trial number
NEO	Neoadjuvant olaparib trial
NEOCATS	Neoadjuvant Olaparib Combination Ovarian Cancer Targeted Study
OPAL-C	OPAL trial, cohort C
PALB2	partner and localizer of *BRCA2*
PAR	Poly(ADP-ribose)
PARP	Poly(ADP-ribose) polymerase
PARP-1	Poly(ADP-ribose) polymerase 1
PDS	Primary debulking surgery
PPM1D	Protein phosphatase Mg^2+^/Mn^2+^ dependent 1D
PRIMA	PRIMA trial
R0	Complete macroscopic resection
RAD51B	RAD51 paralog B
RAD51C	RAD51 paralog C
RAD51D	RAD51 paralog D
SLFN11	Schlafen family member 11
SOLO-1	Study of Olaparib in Ovarian Cancer 1
WoO	Window of opportunity
